# Frequency-dependent viscosity of salmon ovarian fluid has biophysical implications for sperm–egg interactions

**DOI:** 10.1242/jeb.244712

**Published:** 2023-01-13

**Authors:** Marco Graziano, Swomitra Palit, Anand Yethiraj, Simone Immler, Matthew J. G. Gage, Craig F. Purchase

**Affiliations:** ^1^ Department of Biology, Memorial University, St. John's, Newfoundland and Labrador, A1B 3X9, Canada; ^2^ Department of Biological Sciences, Centre for Ecology, Evolution, and Conservation, University of East Anglia, Norwich NR4 7TJ, United Kingdom; ^3^ Department of Physics and Physical Oceanography, Soft Matter Lab, Memorial University, St. John's, Newfoundland and Labrador, A1B 3X7, Canada; ^‡^ Deceased

**Keywords:** Cryptic female choice, Mate choice, Non-newtonian fluids, Ovarian fluid, Sperm competition, Viscoelasticity

## Abstract

Gamete-level sexual selection of externally fertilising species is usually achieved by modifying sperm behaviour with mechanisms that alter the chemical environment in which gametes perform. In fish, this can be accomplished through the ovarian fluid, a substance released with the eggs at spawning. While the biochemical effects of ovarian fluid in relation to sperm energetics have been investigated, the influence of the physical environment in which sperm compete remains poorly explored. Our objective was therefore to gain insights on the physical structure of this fluid and potential impacts on reproduction. Using soft-matter physics approaches of steady-state and oscillatory viscosity measurements, we subjected wild Atlantic salmon ovarian fluids to variable shear stresses and frequencies resembling those exerted by sperm swimming through the fluid near eggs. We show that this fluid, which in its relaxed state is a gel-like substance, displays a non-Newtonian viscoelastic and shear-thinning profile, where the viscosity decreases with increasing shear rates. We concurrently find that this fluid obeys the Cox–Merz rule below 7.6 Hz and infringes it above this level, thus indicating a shear-thickening phase where viscosity increases provided it is probed gently enough. This suggests the presence of a unique frequency-dependent structural network with relevant implications for sperm energetics and fertilisation dynamics.

This article has an associated ECR Spotlight interview with Marco Graziano.

## Teaser

**Summary:** The viscoelastic properties of salmon ovarian fluid support a novel mechanism of post-mating sexual selection in external fertilisers with important implications for understanding the evolution of sexual traits.

## INTRODUCTION

The micro-conditions of fertilisation are poorly understood in the majority of animal species (Cosson, 2015; Eisenbach and Giojalas, 2006; Kholodnyy et al., 2020). Following ejaculation, sperm find and fertilise eggs, but this usually takes place in the presence of post-mating sexual selection arising from sperm competition with rival males (Birkhead and Pizzari, 2002; Parker, 2020) and cryptic female choice that biases paternity (Firman et al., 2017). We now know that polyandry (female mating with multiple males in a given breeding episode) is widespread and common in nature (Taylor et al., 2014) and that post-mating sexual selection plays a crucial role in governing reproductive fitness (Simmons, 2005). It is likely to be responsible for the tremendous diversity in sperm morphology (Ramón et al., 2014; Pitnick et al., 2008) and female reproductive tract morphological complexity (Kelly and Moore, 2016; Sloan and Simmons, 2019). Although many studies have revealed the importance of post-mating sexual selection for dictating variance in individual fertilisation success (Gasparini and Pilastro, 2011; Kekalainen and Evans, 2018; Lüpold et al., 2012), we still understand little about the exact mechanisms that control the outcome of such sexual selection and, ultimately, individual fertilisation success (Birkhead and Pizzari, 2002). In this context, we aimed to explore whether the physical properties of salmon ovarian fluid could support the basis for a physically controlled post-mating choice. Changes in the rheological attributes of the fluid under varying physical stresses might furnish an evolutionary basis for sexual selection in fish, as in other animal models. Specifically, in this paper we analysed the rheological properties of salmon ovarian fluid to explore their role in modulating sperm behaviour and swimming efficiency, looking to highlight a possible novel mechanism of post-mating sexual selection in external fertilisers. Females might be able to sort among sperm from different partners and within a partner, through the governing capacities exerted by their ovarian fluid to favour certain sperm phenotypes, thus influencing their success and evolution.

In terms of female control over paternity, internal fertilisation clearly offers greater direct opportunity to manage sperm and the fertilisation process, compared with external fertilisation. In internal fertilisers, sperm are deposited within the female reproductive tract and then move from the insemination site either directly towards the egg for fertilisation, or indirectly via short- or long-term storage. Sperm can move under their own flagellar propulsion, or be moved by female tract mechanisms, but we rarely understand which sex is controlling sperm dispersal, and how, where and when this occurs throughout the reproductive process. Several female mechanisms could control sperm transfer, progress and activity; from mechanical contractions and hydrostatic pressures in the female tract, to sorting sperm from different males in designated organs and through completely ejecting ejaculates or exerting spermicidal actions (Firman et al., 2017). Biochemical complexity in which these dynamics take place is also important, with evidence that the female tract can be either supportive or, at times, hostile to certain male gametes (Firman et al., 2017; Wolfner, 2011). Ostensibly, much remains to be discovered about this reproductive diversity, with recent *in vivo* research using GFP-tagged sperm revealing high levels of activity and interaction between sperm from different males and different areas of the female tract (Manier et al., 2013a,b).

External fertilisation, in which gametes fuse outside the body in an aqueous environment, appears to present far fewer opportunities for females to exert post-mating control over fertilisation. Gametic interactions cannot benefit from a complex reproductive tract with opportunities for differential sperm uptake, storage and management. However, despite its increased reproductive ‘simplicity’, studies have shown that external fertilisation can indeed allow cryptic female choice via adaptations that encourage the ‘right’ sperm – or discourage the ‘wrong’ sperm – to fertilise (Firman et al., 2017). For example, gamete recognition systems in or on the egg and reproductive fluids, are known to influence sperm behaviour and fertilisation outcome (Evans et al., 2013; Yeates et al., 2013). It is the relative simplicity of these systems compared with internal fertilisers and the tractability of external fertilisation for controlled *in vitro* experiments, that have enabled significant advances in understanding the outcomes and potential mechanisms that control sperm–egg interactions in the context of post-mating selection from sperm competition and cryptic female choice.

Some of our most fundamental knowledge about sperm–egg interactions comes from broadcast-spawning marine invertebrates. The associations between bindin molecules (Palumbi, 1999) and between lysin and its vitelline envelope receptor (VERL) (Swanson and Vacquier, 1997), have been described in detail in sea urchin and *Haliotis*, respectively, where biochemical mechanisms control against the risk of heterospecific sperm attachment or egg membrane penetration (Metz et al., 1994; Palumbi, 1999), influencing individual fertilisation success (Hussain et al., 2016). Similarly, more recent work has described the mechanisms by which female-derived chemoattractants within egg-associated reproductive fluids mediate post-mating mate choice, fertilisation success and offspring fitness in mussels (Fitzpatrick et al., 2012; Oliver and Evans, 2014)*.* In fish, females manufacture ovarian fluid, which is released into the coelomic cavity with maturing eggs (Hirano et al., 1978). Ovarian fluid contains a complex mix of nutrients, metabolites and hormones (Hirano et al., 1978; Ingermann et al., 2001; Lahnsteiner et al., 1995) and once spawned shows the highest concentration in proximity to the micropyle entrance of eggs. The ovarian fluid identity of different females has been found to differentially impact sperm swimming behaviour (Alonzo et al., 2016) and influence fertilisation outcome according to the genetic relatedness of males (Butts et al., 2012; Gasparini and Pilastro, 2011) and their spawning origin (Beirão et al., 2014). In salmonids, ovarian fluid constitutes up to 30% of the spawned egg mass and its influence on sperm is relatively well studied (Galvano et al., 2013; Johnson et al., 2020; Purchase and Rooke, 2020; Turner and Montgomerie, 2002; Zadmajid et al., 2019). There is increasing evidence that this reproductive fluid can act as a ‘fertilisation filter’ for or against sperm from different partners, enabling cryptic female choice. This facilitates sperm selection even in highly polyandrous externally fertilisers like Atlantic salmon (*Salmo salar*), where a single egg batch can be sired by up to 16 fathers (Weir et al., 2010). Yeates et al. (2013) showed that ovarian fluid allowed females to apply conspecific sperm precedence when facing *in vitro* hybridisation risks between Atlantic salmon and brown trout (*Salmo trutta*). However, we do not yet know the exact mechanisms facilitating such choice.

Sperm swimming propulsion is created by the flagellum, whose function is influenced by chemical (Cosson, 2015; Kholodnyy et al., 2020) and physical (Cosson, 2015; Cosson and Prokopchuk, 2014; Holwill, 1977) conditions. The different responses of sperm behaviour reported in the presence of ovarian fluid and their resulting effects on fertilisation (Alonzo et al., 2016; Galvano et al., 2013; Gasparini et al., 2012; Rosengrave et al., 2009), have been associated to changes in pH (Wojtczak et al., 2007), ionic composition (Rosengrave et al., 2009) and viscosity (Turner and Montgomerie, 2002), which together, control flagellar beating (Kholodnyy et al., 2020). While the effects of chemistry (Rosengrave et al., 2009; Wojtczak et al., 2007) and temperature (Dadras et al., 2016; 2017) have been more frequently investigated (Cosson, 2015; Dadras et al., 2016; Kholodnyy et al., 2020), the influence of changes in viscosity on swimming sperm remain poorly explored in external fertilisers (Kholodnyy et al., 2020; Lauga, 2007). There is evidence that fish ovarian fluid possesses structural properties that makes for a non-Newtonian viscous response (where viscosity changes depending on the force applied) that is very different to water (Rosengrave et al., 2009) and this peculiar viscous response could influence the biophysics of sperm swimming behaviour in external fertilisation environments. To describe such function, we conducted detailed measurements of its biophysical characteristics using a rheological approach commonly used in soft-matter physics. We sought to uncover the rheological nature of ovarian fluid when different forces are applied to it, thus exploring how its non-Newtonian behaviour could affect sperm activity, penetration, bioenergetics and guidance to fertilisation in a context of sperm competition and cryptic female choice.

To do this, we collected ovarian fluid from mature females during the reproductive season of Newfoundland wild Atlantic Salmon and analysed its rheological properties. Using a modular compact rheometer (MCR), we applied both steady-state and oscillatory viscosity measurements to determine viscoelastic responses in the non-Newtonian regime, when the ovarian fluids were subjected to increasing shear rates and variable angular frequencies comparable to those exerted by swimming spermatozoa moving in the fluid toward the eggs. By evidencing specific shear-dependent changes in the polymeric structure of the fluid and how these occur, we expected to identify an underlying mechanism that influences partner selection to provide an evolutionary advantage.

## MATERIALS AND METHODS

### Sample collection and preliminary measurements

Wild anadromous Atlantic salmon (*Salmo salar* Linnaeus 1758) were collected in early September from a fish ladder at Grand Falls (48° 55′ N, −55° 39′ W) during their up-stream spawning migration on the Exploits River (Newfoundland, Canada). Following previous protocols (Rooke et al., 2019), fish were transferred to covered, outdoor tanks next to the river and experienced ambient temperatures and light. Over two weeks in early November, females were assessed for ovulation using gentle abdominal pressure, fish were then anaesthetised using a solution of 0.2 ml l^−1^ clove oil, measured for length, weighed and stripped of eggs after drying the urogenital pore. Each female's eggs (and associated ovarian fluid) were kept in sealed glass jars, enclosed with bubble wrap and placed in a cooler of wet ice for transport to the laboratory. Each egg batch was separated from its ovarian fluid using a fine mesh net (Purchase and Rooke, 2020) within 10 h of stripping. For each ovarian fluid sample we recorded volume and mass to deduce density, followed by pH and conductivity.

### Rheological characterisation of ovarian fluid

The mechanical properties of many soft biological materials are neither purely viscous (liquid-like) nor purely elastic (solid-like) and these rheological properties correlate strongly with their function (Storm et al., 2005). Structured fluids often do not flow until they reach a critical stress level, below which a material is considerable elastic and above which the structure of the material breaks down and starts to flow. Two experiments were performed to define how the ovarian fluid's polymeric structure (and related physical properties that in turn would affect sperm swimming activity) can be modulated, depending on swimming sperm flagellar beat frequency. Specifically, we tested ovarian fluid ‘behaviour’, both under steady shear (i.e. ‘flow curves’) and under small-amplitude oscillatory shear (SAOS). The former examines the viscoelastic response of the ovarian fluid by continuous deformation and breakup of internal networks, while the latter can probe weaker internal structures (Ferry and Myers, 1961; Goddard, 1979). A preliminary rheological analysis (*n*=5 fish) was conducted to assess different fluid preservation methods (see Supplementary Materials and Methods). Each frozen sample was thawed at room temperature for 1 h prior to analysis and measurements were made using 1.5 ml aliquots. All the analyses were performed in the Soft Matter Lab at Memorial University using an MCR 301 rheometer, equipped with a cone-plate (CP50-0.5, 50 mm diameter plate and cone angle, Anton Paar, St Albans, UK) system. Ovarian fluid samples were individually filtered through a 200 µm sieve to remove any particulates (e.g. coagulated blood, ovarian tissue) that could influence the rheological measurements. Pipetted fluid was equilibrated for three minutes at the plate temperature of 6°C, allowing for homogenous sample relaxation from any uncontrolled pre-shear imposed on the fluid during loading. The temperature of 6°C was chosen because it resembled the natural water temperature experienced by Atlantic salmon in the Exploit River at spawning (see also Rooke et al., 2019).

### Steady-state shear properties

Samples were tested for their resistance to flow in order to measure their viscosity under a specific rate of deformation. To obtain a flow curve, the shear stress was measured for a range of shear rates (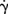), from 10 to 500 s^−1^ in 50 equally spaced steps. The resultant shear stresses of the ovarian fluid were measured to determine the apparent viscosity η_a_, which was averaged across three aliquots per female (*n*=11) and plotted as a function of the shear rate.

Among each of the three ovarian fluid aliquots per fish, a run with distilled water was performed as a control. For distilled water (pure Newtonian fluid), a theoretical positive relationship between shear stress and shear rate should be linear and the fit line should pass through zero. When the profiles of water runs were fitted, a positive intercept (typical for these kind of measurements) of 0.0133 Pa was concluded to be low shear rate instrumental noise. It was subtracted from all the water and ovarian fluid samples for standardisation [(shear stress–0.0133 Pa)/shear rate], creating a small change in values. A comparison of individual ovarian fluid viscosity profiles with distilled water for each of the instrumental replicates allowed us to assess variability among females.

The apparent viscosity of ovarian fluid decreased with increasing shear rates, in contrast with water whose apparent viscosity (η_a_=0.00151±0.00003 Pa s) was independent of shear rate. The apparent viscosity at 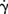
=10 s^−1^ was roughly 10 times the viscosity of water, but returned within comparable values under increasing shear rates, starting at around 100 s^−1^ (see Results). For three females the ovarian fluid samples had apparent viscosities η_a_ in the order of 0.003 Pa s at 10 s^−1^, showing no meaningful differences with the rheological behaviour of water at the same shear rate. These samples were probably contaminated with urine and/or water during stripping of gametes and for these reasons were not included in the main results. The remaining 11 flow curves were globally fitted to the form η_a_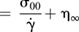, which is a simple equation incorporating an elastic component, the yield stress σ_00_, which must be overcome before there is flow and a viscous component η_∞_, which represents the viscosity at very high shear rates. This simple form was arrived at when fitted to a more complicated formula, the Herschel–Bulkley equation 
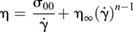
 (Herschel and Bulkley, 1926) resulted in power laws *n* that were very close to unity.

### Small-amplitude oscillatory sweeps

To preserve finer polymeric structures and obtain a dynamic profile that informs about the viscous and elastic components, we subjected the ovarian fluid to small-amplitude oscillatory shear. For these measurements, a sinusoidal deformation (γ=γ_0sin_ *t*) was imposed on the sample at a fixed frequency (ω) and a maximum amplitude (γ_0_) (Schoff and Kamarchik, 2005). Measurements were performed for a range of ω, from 0.01 to 500 rad s^−1^ in 24 equally spaced logarithmic increments (strain amplitude 5%), where ω is defined as ω=2 π *f*, where *f* is the frequency in Hz. The storage modulus:
(1)

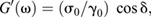
and the loss modulus:
(2)

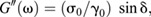
where σ_0_ is the oscillatory stress applied and δ is the phase angle, were obtained as a function of frequency (ω). The modulus of the complex viscosity η* was obtained from the relation:
(3)

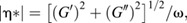
while the damping factor (or loss factor) tan δ ≡ *G″*/*G′* represents the ratio between viscous and elastic contributions to the viscoelasticity.

### Applicability of the Cox–Merz rule

The Cox–Merz rule, an empirical method to rationalise steady shear and oscillatory rheological data (Cox and Merz, 1958), was used to compare the two different rheological analyses adopted in our study. A strong correlation between two independent methodologies is a good consistency check. This rule states that the apparent viscosity (η_a *=*_σ/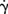) at a specific shear rate (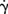) is equal to the complex viscosity (|η*(ω)|=|G*(ω)|/ω) at a specific oscillatory frequency (ω), that is:
(4)

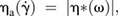
with 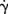 = ω. When the rule is obeyed, rheological properties of a fluid can be described by either oscillatory or steady-state shear experiments (Rao et al., 2014).

### Statistical analyses

All ovarian fluid measurements and fish morphological data [mean±s.d., 95% CI and coefficient of variation (CV%)] were summarised using the descriptive statistics function in GraphPad Prism, v. 8.0.0, (GraphPad Software, San Diego, USA). Rheometer reads were first standardised for instrumental error and the model fits were applied as described above. Subsequently, the average values of *G′* and *G″* (dependent variables) across all the sampled females were pair-wise compared through *t*-tests at specific frequencies (independent variables) of interest within two shear stress ranges, 0.001 to 0.105 and 0.105 to 1 rad s^−1^, to confirm their uniformity within the plateau region and/or alternatively the prevalence of either the viscous or the elastic component of the ovarian fluid in this dimensional range. Normality of the residuals was ensured by using the D′Agostino–Pearson test followed by Shapiro–Wilk test (*P*=0.2174 and 0.4697, respectively). In all analyses, the statistical significance threshold used was α=0.05.

## RESULTS

**Fig. 1.**
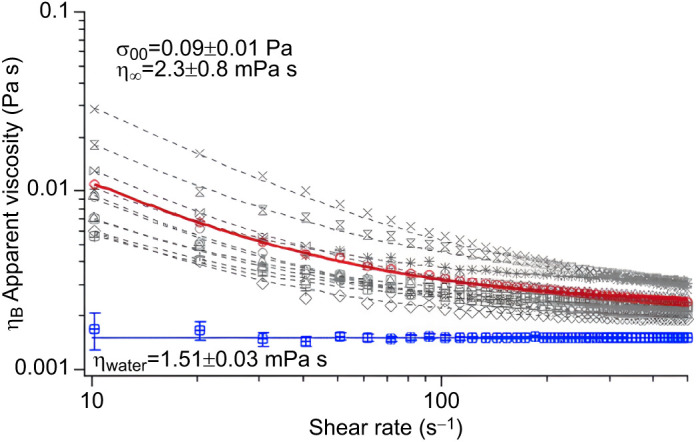
**Apparent viscosity obtained from the steady shear flow curves of Atlantic salmon ovarian fluid.** Shear flow curves (η) of ovarian fluid samples (*n*=11, in grey) and water controls (in blue), plotted versus shear rate (s^−1^) on a log–log scale. Grey symbols and dotted lines represent individual ovarian fluid means across 3 replicates per female and their fitted equations, respectively, while the red and blue symbols and the continuous lines represent the mean across all ovarian fluid samples (red) and water controls (blue). The symbols σ_00_ and η_∞_ are, respectively, the yield stress and the apparent viscosity at high shear rates obtained from fitting to the Herschel–Bulkley equation; η_water_ instead represents the average apparent viscosity value of water within the analysed shear rates (mean±s.d.).

Ovarian fluid characteristics varied among individual females (Table 1). For context, coefficient of variation [(s.d./mean) ×100] of fish length was 10% while body mass (which included eggs and ovarian fluid) was 34%. The amount of ovarian fluid produced for a given size of fish or mass of eggs was very inconsistent among females (CV ∼50%). Conversely, fluid density, pH and conductivity were similar (<10% and thus less variable than fish length). Apparent viscosity was highly variable among fish, but all exhibited clear non-Newtonian behaviour. The amount of variation declined with the shear rate applied, being CV=57% among females measured at 10 s^−1^ and CV=17% at 500 s^−1^ (Table 1, Fig. 1).

**Table 1.**
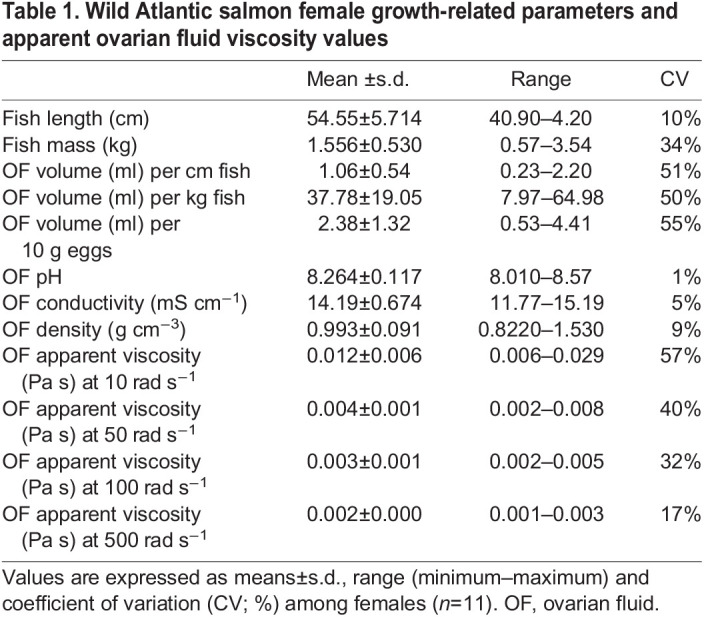
Wild Atlantic salmon female growth-related parameters and apparent ovarian fluid viscosity values

### Ovarian fluid rheology in steady-state shear flows

To measure the viscosity under a linearly increasing rate of deformation, the ovarian fluid samples were tested for their resistance to flow for a range of shear rates (10 to 500 s^−1^). The resulting shear stress responses from the deformed ovarian fluid were measured to determine the apparent viscosity of the material at each of the measuring points.

Atlantic salmon ovarian fluid showed non-Newtonian shear-thinning behaviour indicating successive loss of polymer entanglements with increasing shear rates (Fig. 1). The Herschel–Bulkley equation fits returned a mean value of yield stress σ_00_=0.09 (± 0.01 Pa) and a mean value of the high shear viscosity η_∞_=2.3 (± 0.8 mPa s) with the ovarian fluid showing an average 97% decline in viscosity as an increasing shear rate was applied through the rheometer's plate (Fig. 1).

### Small-amplitude oscillatory sweeps and dynamic shear properties of the ovarian fluid

**Fig. 2.**
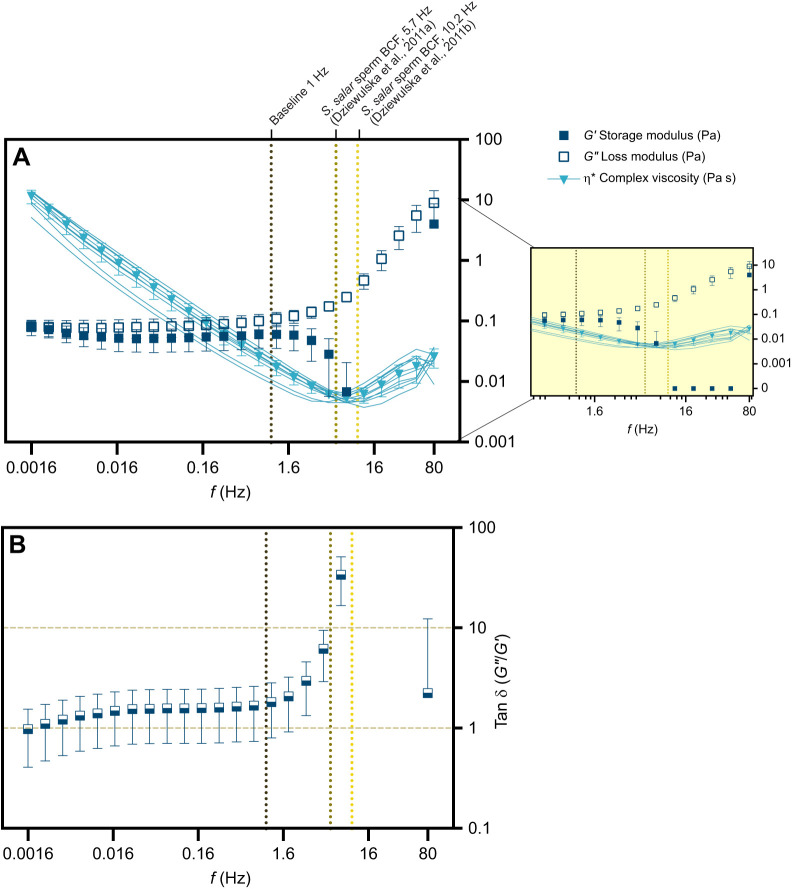
**Relationship between the viscous and elastic components of Atlantic salmon ovarian fluid at increasing angular frequencies.** (A) Storage modulus (*G*′), loss modulus (*G*″) and complex viscosity (η*) of ovarian fluid (*n*=11), at increasing angular frequencies (0<Ω<500 rad s^−1^, or 0.0016 to 80 Hz for an easier comparison with sperm beat cross frequencies). Data are presented as means±s.d., continuous lines for η* represent individual ovarian fluid means across three replicates for each female; the data points not shown in the main graph (please note the log–log scale) are shown in the inset on the right. (B) Loss factor (tan δ=*G*″/*G*′) of Atlantic salmon ovarian fluids (means±s.d.) plotted versus frequency (Hz), where tan δ=100 for a liquid material with a pure viscous behaviour and tan δ=0.01 for a solid material with an ideally elastic behaviour. Vertical dotted lines from left to right represent a reference baseline at 1 Hz and Atlantic salmon average sperm tail beat frequencies in Hz from Dzievulska et al., 2011a, 2011b. Graphs are plotted on log–log scale.

The dynamic viscoelastic behaviour of the ovarian fluid dispersions was also determined by applying SAOS frequency sweep. The storage modulus *G*′ and loss modulus *G″*, shown in Fig. 2(A), were not different at low frequencies, with both having a value of approximately 0.1 Pa in the 5 measuring steps between 0.01 and 0.105 rad s^−1^ [0.065±0.011 Pa and 0.077±0.001 Pa, respectively (mean±s.d.); *P*≥0.05, *t*=2.77, d.f.=4] and describe a pure viscoelastic fluid where the elastic and the viscous components of the fluid are comparable. Both *G′* and *G″* decreased slightly between 0.01 and 0.07 rad s^−1^ (note the log_10_–log_10_ axes) and thereafter maintained constant plateau values until the shear rate reached 1 rad s^−1^. Note that this plateau value is numerically proximate, given the errors, to the value obtained for the yield stress in the steady shear measurements. Salmon ovarian fluid is therefore a gel-like structure at low frequencies and becomes more dominantly liquid-like at frequencies higher than 10 Hz. Interestingly, this structural shift occurs in a dimensional range that overlaps with the frequencies exerted by salmon sperm when swimming through the ovarian fluid to reach the egg (refer to dashed vertical lines in Fig. 2A,B). This is confirmed also by the fact that at low frequencies, the gel-like structure is supported by a value of tan δ =*G″*/*G′* of 1 (crossover or gel point, see Fig. 2B); however, between frequencies of 0.10 to 1 rad s^−1^ (6 steps) the loss modulus *G″* (mean 0.081±0.02) was marginally higher (*P*<0.001, *t*=32.93, d.f.=5) than *G′* (0.052±0.01). As observed through the study of their first and second derivatives, *G′* and *G″* trends start to slowly diverge, more intensely from 10 Pa (at 1.59 Hz) onward revealing a breakpoint in the polymer that exacerbates together with increasing shearing rates (supplementary Materials and Methods, Figs S1 and S2). Specifically, the storage modulus reached 0 Pa between 47.6 and 312 rad s^−1^ (7.58 and 49.66 Hz), showing that the elastic response of the polymer under these frequencies is null (liquid-like); and viscous forces at their maximum in this frequency range instead prevailed. As a result, the absolute value of the complex viscosity (|η*|) decayed until reaching its a minimum of 0.005 Pa s, at a frequency near 8 Hz (50 rad s^−1^), (see Fig. 2A). Interestingly, |η*| increased after this measuring point. Values of tan δ =*G″*/*G*′, were similar at low frequencies and also showed a clear dependence in the same frequency range increasing to 34±17 at the highest frequencies.

### Comparison of steady and oscillatory shear

**Fig. 3.**
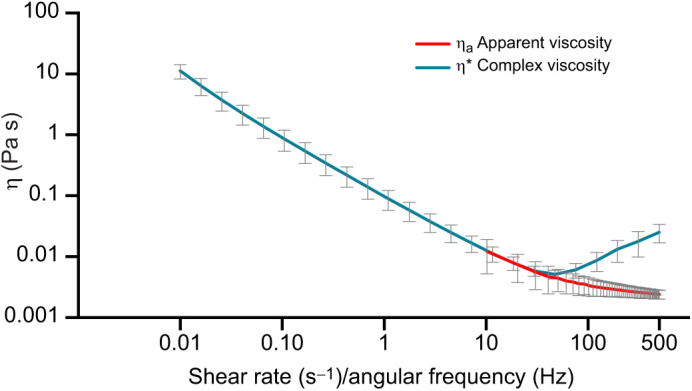
**Comparison between steady state and the dynamic properties of Atlantic salmon ovarian fluid.** Apparent viscosity obtained from the steady shear flow curves (η_a_) of ovarian fluid samples (*N*=11, red) and complex viscosity (η*) of ovarian fluids (*n*=11, blue) plotted versus shear rate (s^−1^) and angular frequency (Hz). Values are presented as means±s.d. Graph is plotted on log–log axes.

The steady-state properties of the ovarian fluid were compared with the dynamic states by applying the Cox–Merz rule. This rule, applied to polymers, enables the identification of secondary flow behaviours and/or breaking down of the fluid's polymeric network under a certain imposed stress. Apparent viscosities (η_a_) obtained in the flow curves and absolute values of complex viscosities (|η*|) resulting from the small-amplitude oscillatory sweep experiments, were plotted as a function of shear rate and angular frequency, fitted to the best trend and assessed for deviations between the curve profiles (Fig. 3). Ovarian fluid η and η* followed the same trend with many remarkable similarities. When the oscillatory shear probed lower frequencies, the curves overlapped very closely. From the steady shear results, we extracted a yield stress σ_00_=0.09±0.01 Pa, which is close to the *G′* plateau value of σ_0_=0.068±0.006 Pa. However, above 50 rad s^−1^ (8 Hz), there is an increase in |η*|.

Beyond this frequency, the Cox–Merz rule was not obeyed, meaning that η_a_ and |η*| values obtained at a specific shear rate are not equal when compared between the two different methodologies used. It should be noted that steady shear is much more disruptive to the gel structure than oscillatory shear. Thus, while we must be cautious with interpreting the rise in |η*| between 50 and 500 s^−1^, it is nevertheless feasible that this rise is indicative of a rise in the SAOS viscosity.

## DISCUSSION

We describe the rheological characteristics of Atlantic salmon ovarian fluid to understand the possible involvement in sexual selection mechanisms. We subjected ovarian fluid from different females to both variable shear stresses (steady-state rheology) and angular frequencies (SAOS), that were similar to those exerted by sperm swimming through ovarian fluid to fertilise eggs (Dziewulska et al., 2011a,b). This allowed for the identification of the main viscoelastic profile of the fluid, but also for inferring secondary flow behaviours and the eventual breaking down of macromolecular entanglements under a certain imposed stress. In particular, SAOS measurements describe the viscous and elastic components within the ovarian fluid that could affect fertilisation dynamics. We found that the physical characteristics of salmon ovarian fluid clearly show a non-Newtonian viscoelastic nature, where shear-influenced changes in viscosity and elasticity might have the potential to influence fertilisation. Here, we discuss the structural characteristics of the ovarian fluid that could influence sperm and explore the potential of its non-Newtonian properties to be adaptive.

### Shear-thinning behaviour in steady-state rheology and under small-amplitude oscillatory sweeps

Our results indicate that ovarian fluid, which is a gel at its relaxed state (between solid- and liquid-like behaviour), is a shear thinning viscoelastic liquid at low frequencies and may exhibit a shear thickening phase at high frequencies. This shift from gel to a more passable medium, together with minimum viscosity values observed within the range of average beating frequencies exerted by salmon sperm, points out an interesting overlap that might be linked to ‘bio-mechanical co-evolution’ of female and male gametes. Specifically, Atlantic salmon ovarian fluid has a viscosity at its relaxed state that is on average 60 times that of water, being 0.09 Pa. A hypothetical beating frequency of 1 Hz would yield to the absolute value of the complex viscosity |η*| of 0.017 Pa s (five times lower than at its relaxed state), while a beating frequency of 12 Hz yields 0.006 Pa s. Sperm movement occurs at low shearing rate (Brokaw, 1965; 1966) and the sperm beat cross frequency (BCF) values reported in the literature (∼5–10 Hz) (Dziewulska et al., 2011b,a) are in a similar range of frequencies as used in our experiment. Fascinatingly, these frequencies correspond to either the shear thinning region or to the minimum values of apparent and complex viscosity reported, having a meaningful biological translation. Another intriguing possibility is that the departure from the Cox–Merz rule at high frequencies might actually signal an increase in viscosity when measured in a sensitive way (SAOS) that does not disrupt gel structure, but not when measured in a more disruptive manner (steady shear). There might therefore even exist an optimal beat frequency window below and above which the ovarian fluid is effectively more viscous.

Flagellar beating frequency varies considerably with temperature, pH, time, activation medium (e.g. water vs. ovarian fluid) and methodology used to detect it (Cosson, 2021; Zadmajid et al., 2019). Measures from other salmonids obtained at higher temperatures, in a diluted solution of ovarian fluid and using stroboscopic techniques, show higher frequencies, such as ∼50 Hz for *Oncorhynchus mykiss* (Cosson et al., 1985) and ∼80 Hz for *Oncorhynchus tshawytscha* (Butts et al., 2017). For this reason, we will more cautiously consider for this discussion a broader range for sperm beating frequency of salmon sperm in ovarian fluid up to 80 Hz.

Our viscosity measures are considerably higher than reported for other fish species, such as 0.0038 Pa s (2.76 times that of water) for Arctic charr (*Salvelinus alpinus*) when measured at 0.5 Hz under a plate viscosimeter (Turner and Montgomerie, 2002). In chinook salmon (*O. tshawytscha*) ovarian fluid viscosity decreased from 0.0042 to 0.0027 Pa s as shear was increased from 7 to 72 Hz (Rosengrave et al., 2009). These lower values reported in other species might be related to the higher starting frequency used as compared to ours. In fact, if paralleled to what we found in *S. salar*, the starting point of 7 Hz used for *O. tshawytscha* falls within the shear thinning phase of the fluid, implying that this was first probed already under a certain initial stress rather than at its relaxed state, thus masking a potentially higher relaxed state viscosity. In our case, by controlling for instrumental uncertainty and comparing two different rheological approaches, we had the advantage of precisely probing and extracting realistic zero shear viscosities and low shear values. This is relevant because our results not only show that the gap in viscosity caused by increased shear is greater than previously thought, but so will be the biological implications resulting from different frequencies shearing the fluid.

### Viscous and elastic components within the ovarian fluid that could affect fertilisation dynamics

Viscous compounds are known to influence movement of the flagellum, resulting in a lower velocity (Brokaw, 1965). Brokaw (1966, 1983) investigated sperm flagellar behaviour in response to increased viscosity in three marine Phyla (Anellida, Tunicata and Echinodermata), finding a decrease of both beat frequency and wavelength, similar to what was found in chinook salmon (Butts et al., 2017). These authors partially justified an observed increase in velocity and propulsive efficiency of sperm swimming in ovarian fluid through the non-Newtonian properties of this medium. These were firstly described in a study by Rosengrave et al. (2009), who explored its response to shear rates under a constant rotational force (steady-state properties). By including both steady-state measurements and SAOS, we add crucial information on the specific elastic and viscous components within the ovarian fluid that could justify the changes in sperm behaviour reported by other authors and further investigate its role during reproduction.

In view of our rheological results which show viscoelastic behaviour of salmon ovarian fluid, new considerations need to be made because the viscous (liquid-like) and elastic (solid-like) components of the fluid define its changing complex viscosity and cannot be neglected when analysing sperm energetics and outcome. Lauga (2007), has proposed that the non-Newtonian properties of a biological fluid (cervical mucous of internal fertilisers in that work), might allow it ‘to tune passively transport kinematics by modulating material properties’, making them advantageous in selecting the appropriately motile spermatozoa; such characteristic is instead missing in Newtonian fluids (Lauga, 2007). More recent works have summarised the different responses of micro swimmers in complex viscoelastic environments and under different flows (Lauga, 2020; Li et al., 2021).

In studies with internally fertilisers (mammals), viscoelastic reproductive fluids have been found to decrease spermatozoa velocities as viscosity increased. However, this was associated to a concurrent increase in their linearity (Suarez and Pacey, 2006). *Bos taurus* have higher thrust efficiencies of sperm when swimming in a non-Newtonian fluid rather than in a Newtonian one, which could be due to a better energetic exploitation of the elastic responses of the fluid (Hyakutake et al., 2019). Similarly, computational models of undulatory swimmers have shown a speed-up effect due to elasticity; although this boost was present only when associated to an asymmetric stroke of the undulation (Thomases and Guy, 2014). In our case, we observe a drop in absolute value of the complex viscosity as the frequency is increased up to 8 Hz (absolute viscosity minimum) when subjecting salmon ovarian fluids to SAOS, suggesting that until this point sperm find an increasingly thinner polymeric network that gets looser with frequency. This happens first in presence of a good elastic component that instead collapses in the ‘armpit region’, having the potential to positively influence sperm linearity and guidance. In this fluid, sperm with different tail beating frequencies, would in principle face substantially different polymeric structures within the shear-thinning phase. This shear-thinning flow behaviour could either facilitate sperm penetrating the egg, or it could also enable cryptic female choice if a specific sperm, its morphological phenotype, swimming behaviour or another trait, is favoured over the one of a rival male competing to fertilise the eggs. Moreover, if considering the reported within-male sperm variability observed in *S. salar* (Immler et al., 2014), it is presumable that the physical properties of ovarian fluid might have a role also in within-male sperm selection. Cryptic female choice between individual males, has recently been identified as a neglected component of post-mating sexual selection; the incorporation of such mechanisms operating selection among different sperm haplotypes and phenotypes within the same ejaculate into models of sexual selection may broaden the current knowledge of the selective pressures driving the evolution of mating systems (Kekäläinen, 2022).

Sperm traits are under strong selection (Fitzpatrick et al., 2020; Fitzpatrick and Lüpold, 2014), with recent studies evidencing a relation between some sperm traits and offspring fitness (Immler et al., 2014) and a correlation between sperm phenotype and genotype (Alavioon et al., 2017). Moreover, sperm within the same ejaculate can experience different stressors that negatively affect their swimming behaviour; the impairment of these ‘abnormal’ gametes is also reflected on a molecular level (e.g. DNA fragmentation) (Fernández et al., 2003), influencing the quality of the information transmitted to the zygote and accordingly its performance. Flagellar activity declines with time post-activation, while the osmotic- and ROS-derived damage experienced by the sperm cell increases (Kholodnyy et al., 2020). Therefore, the peculiar non-Newtonian properties of this fluid, shear-thinning at low shear rates, followed possibly by shear-thickening, might help select the best performing sperm within a single ejaculate, with the objective of limiting the chance of ‘abnormal’ sperm from penetrating the eggs. The frequency-dependent minimum in viscosity, raises therefore the intriguing possibility that the ovarian fluid selects for an optimal speed, providing a viscosity cost for both slow and fast beating sperm. Increasing the swimming cost for very fast sperm/fertilisation could eventually allow selection based on further biochemical mechanisms that are pivotal for sperm–egg interaction, can influence the reproductive outcome and have been suggested to reduce the hybridisation risk with other species (Yanagimachi et al., 2017).

It is well accepted that the guidance within reproductive fluids occurs by means of chemical and biochemical cues that can differentially enhance the reproductive outcome from different males as demonstrated in a range of external fertilisers (Cosson, 2015; Cosson et al., 2008; Evans and Lymbery, 2020; Evans and Sherman, 2013; Kholodnyy et al., 2020; Yanagimachi et al., 1992; Zadmajid et al., 2019).

We propose that more consideration should be given to the physical characteristics of the ovarian fluid that could affect sexual selection processes. Females might be able to facilitate the progression of the high quality and fast beating sperm, within and among ejaculates. Also, in view of the variability observed across females, they might have different capabilities to exert this selective potential and such potential could change with the hydration grade of the ovarian fluid as the reproductive season advances.

### Shear-thickening behaviour

Under SAOS at the highest shear rates measured, we observed a significant stiffening of the polymer. This did not occur in steady-state measurements, where the fluid continued to thin up to 80 Hz. This difference could identify the presence of weak network associations that are broken in steady-state flow measurements, where a continuous rotational force is applied on the fluid. In contrast, these interconnected networks are unaffected in the oscillatory shear tests. In this case, the ovarian fluids were subjected to sinusoidal shear stresses within a linear range small enough that the macromolecular entanglements are preserved. The storage modulus in the shear rates of interest appears to be between 0.1 and 1 Pa, compatible with values of typical network-like structures such as cell suspensions and cell lysates, having storage moduli in the order of 1 Pa (Ballica et al., 1992; Shi et al., 1993). Rheological measurements on the roe of Alaska walleye pollock (*Gadus chalcogrammus*) showed values between 1 and 10 Pa (Anvari et al., 2018), so this appears consistent.

A shear thickening phase at very high frequencies, mostly in absence of any elastic component, would suggest that sperm swimming efficiency could be exclusively dependent on its speed and on the fluid viscosity, without exploiting the positive effects on linearity that some elasticity would provide. The lack of elasticity on the other hand may also be promoting more pronounced circular trajectories, rather than linear ones, which in a closely related species (*O. mykiss*) has been linked to augmenting the chances of fertilising the egg (Wojtczak et al., 2007).

It could be further speculated that a shear-thickening phase at high frequencies might also be linked to the ‘necessity’ for ovarian fluid to stay close to the eggs and not be washed away – an aspect of natural selection. Salmon spawn in rivers and an infinitely shear-thinning ovarian fluid would enhance its chances of being dispersed and diluted very quickly, thus depriving the eggs of its known beneficial effects on fertilisation (Alonzo et al., 2016; Butts et al., 2012; Gasparini and Pilastro, 2011; Poli et al., 2019; Yeates et al., 2013). Other works have shown that a shear-thickening behaviour observed at high frequencies could be also derived from inertial forces. For example, a study of hagfish slime (Böni et al., 2016) presented similar behaviour at low frequency with *G″*/*G′*≈1, indicating an ultra-soft material with weak elastic properties. However, in that study a rise in *G″* (and drop in *G′*) at higher frequencies was attributed to instrument inertia. We cannot exclude that this could also be the case here. Moreover, although the idea that the ovarian fluid may have a natural selective function at very high shear rates is indeed fascinating, this specific aspect lies outside the scope of this study and was not tested specifically. Future experiments should try to provide insights in this regard by testing the ovarian fluid dispersion capacity from eggs under different shear rates that could better simulate the riverbed waterflow that characterises the reproduction of most salmonids. Also, until the shear thickening behaviour of the ovarian fluid is confirmed, the complete picture of how sperm swim in ovarian fluid is not clear. Currently, all the modelling approaches published, have assumed exclusively a shear-thinning fluid at smaller shear flows such as those experienced by sperm in the genital tract of internally fertilising animal models (Guasto et al., 2020; Kumar and Ardekani, 2020; Simons and Rosenberger, 2021). In fact, recent findings have evidenced how the elliptical trajectories of the sperm change as a consequence of different external shear flows and with changing ratios between the elastic and the viscous predominancies within the fluid, where the length and the stiffness of the flagellum can have profound influence on its swimming efficiency (Kumar and Ardekani, 2020).

### Concluding remarks and future perspectives

Ovarian fluid physical properties deserve more attention and considerations when studying processes of sexual selection such as selection on sperm performance, sperm competition assays and fertilisation trials, both *in vivo* and *in vitro*. The characteristic rheological behaviour of the ovarian fluid we report here underlines the importance of including it as a preferred sperm activation medium over pure water to simulate a more natural fertilisation environment and benefit from its effects on sperm.

Notably, our findings suggest that processes enabled by non-Newtonian reproductive fluids within female internal genital tracts, like lubrication, facilitation and capacitation, should also be applied to the external fertilisation environment. This opens new avenues into the study of cryptic female choice with important implications for understanding the evolution of sexual traits and exploring the underestimated role of physical properties of the fertilisation environment that surrounds the gametes both in nature and in artificial fertilisation protocols.

Our discovery yields a number of predictions to be tested in the future, including testing whether the physical properties of ovarian fluid act as a filter for specific sperm or, whether its structure only ameliorates sperm performance in general. Moreover, how higher and/or turbulent shear flows could influence external fertilisers and in particular aquatic spawners among them, should still be clarified.

Further studies should test whether the shear-thickening phase observed at the upper end of our analysed range is trustworthy and if this persists at very high frequencies with a beneficial effect on the eggs (e.g. higher diffusion, mechanical resistance, pathogen barrier) (Elofsson et al., 2003).

## Supplementary Material

10.1242/jexbio.244712_sup1 Supplementary information
